# LtrDetector: A tool-suite for detecting long terminal repeat retrotransposons de-novo

**DOI:** 10.1186/s12864-019-5796-9

**Published:** 2019-06-03

**Authors:** Joseph D. Valencia, Hani Z. Girgis

**Affiliations:** The Bioinformatics Toolsmith Laboratory, Tandy School of Computer Science, University of Tulsa, 800 South Tucker Drive, Tulsa, 74104 OK USA

**Keywords:** Long terminal repeats retrotransposons, Repeats, Software, Signal processing

## Abstract

**Background:**

Long terminal repeat retrotransposons are the most abundant transposons in plants. They play important roles in alternative splicing, recombination, gene regulation, and defense mechanisms. Large-scale sequencing projects for plant genomes are currently underway. Software tools are important for annotating long terminal repeat retrotransposons in these newly available genomes. However, the available tools are not very sensitive to known elements and perform inconsistently on different genomes. Some are hard to install or obsolete. They may struggle to process large plant genomes. None can be executed in parallel out of the box and very few have features to support visual review of new elements. To overcome these limitations, we developed LtrDetector, which uses techniques inspired by signal-processing.

**Results:**

We compared LtrDetector to LTR_Finder and LTRharvest, the two most successful predecessor tools, on six plant genomes. For each organism, we constructed a ground truth data set based on queries from a consensus sequence database. According to this evaluation, LtrDetector was the most sensitive tool, achieving 16–23% improvement in sensitivity over LTRharvest and 21% improvement over LTR_Finder. All three tools had low false positive rates, with LtrDetector achieving 98.2% precision, in between its two competitors. Overall, LtrDetector provides the best compromise between high sensitivity and low false positive rate while requiring moderate time and utilizing memory available on personal computers.

**Conclusions:**

LtrDetector uses a novel methodology revolving around k-mer distributions, which allows it to produce high-quality results using relatively lightweight procedures. It is easy to install and use. It is not species specific, performing well using its default parameters on genomes of varying size and repeat content. It is automatically configured for parallel execution and runs efficiently on an ordinary personal computer. It includes a k-mer scores visualization tool to facilitate manual review of the identified elements. These features make LtrDetector an attractive tool for future annotation projects involving long terminal repeat retrotransposons.

**Electronic supplementary material:**

The online version of this article (10.1186/s12864-019-5796-9) contains supplementary material, which is available to authorized users.

## Background

Formerly considered “junk DNA”, the intergenic sequences of genomes are attracting increased attention among biologists. A particularly striking feature of these regions is the prevalence of transposable elements (TEs), a type of repeated sequence. TEs include class I elements, which replicate using RNA to “copy-and-paste” themselves, and class II elements, which replicate via a “cut-and-paste” mechanism using DNA as an intermediate [1]. Barbara McClintock discovered transposons in the 1940s and the 1950s while studying the maize genome [2]. TEs are common to all eukaryotes, comprising around 45% of the human genome and up to 80% of some plants like maize and wheat [3, 4].

TEs have several important functions. *Bennetzen and Wang* highlight the known functions of plant TEs [5]. Transposons are the major factor affecting the sizes of plant genomes [6–8]. Under stressful conditions, they can rearrange a genome [9–11]. TEs play roles in relocating genes [12, 13] and generating new genes [14, 15] and new pseudo genes [16, 17]. They can contribute to centromere function [18, 19]. TEs can regulate the expression of nearby genes via several mechanisms including: (i) providing regulatory elements, such as promoters and enhancers, to nearby genes [14, 20–22]; (ii) inserting themselves into genes, then targeting the epigenetic regulatory system [23]; (iii) producing small interfering RNA specific to host genes [24–26]; and (iv) generating new micro RNA genes modulating host genes [27–29]. Transposons have been utilized in cloning plant genes in a technique called transposon tagging [30–32]. They also have the potential to become a new frontier in enhancing the productivity of crops [33, 34].

Long Terminal Repeat retrotransposons (LTR-RTs) are a particularly interesting type of class I transposable element related to retroviruses. LTR-RTs are widespread in plants and are considered one of their primary evolutionary mechanisms [35]. *Gonzalez*, et al. summarizes some of their functions [36]. LTR-RTs can insert adjacent to and inside of genes and promote alternative splicing [37], They play roles in recombination, epigenetic control [38, 39], and other forms of regulation [36]. LTR-RTs have been found with regulatory motifs that promote defense mechanisms in damaged plant tissues [40]. They can also serve as genomic markers for evolutionary phylogeny [41].

LTR-RTs are named for their characteristic direct repeat — typically 100–6000 base pairs (bp) long in plants. These direct repeats surround interior coding regions (the *gag* and *pol* genes). Lerat suggests 5 kbp–9 kbp as a size range for LTR-RTs [1], but based on the consensus sequences of plant LTR-RTs, their lengths can exceed 20 kbp.

Computational tools are extremely important in locating repeated sequences, including LTR-RTs. Tools can be roughly divided into knowledge-based tools, which leverage consensus sequence databases to search for repeats, and de-novo tools, which use internal sequence comparison and structural features to search for repeats without prior knowledge about the target sequence [1].

Knowledge-based methods include well-known bioinformatics software such as NCBI BLAST [42], RepeatMasker (http://www.repeatmasker.org), and Censor (https://www.girinst.org/downloads/software/censor/); they can be utilized in locating all types of known TEs including LTR-RTs. However, if the sequence of the repetitive element is unknown, tools like these cannot find copies in a genome.

Several methods for locating all types of TEs de-novo have been developed [43–46]. Tools built specifically for detecting LTR-RTs include LTR_STRUC [47], LTR_seq [48], MGEScan-LTR [49], LTR_Finder [50], and LTRharvest [51]. LTR_retriever is a post-processing tool, which may help increase the accuracy of de-novo approaches [52]. LTRsift [53] and Inpactor [54] are other post-processing tools that cluster LTR-RTs into families and allow additional analyses.

These tools face a variety of usability, scalability, and accuracy concerns. For example, LTR_STRUC, one of the pioneering tools for locating LTR-RTs, was developed exclusively for an old version of Windows, making it difficult to use nowadays. Several tools have external dependencies which greatly complicate their installation. None of them take advantage of the parallel multi-core architecture of modern personal computers. Some may struggle to process larger plant genomes such as the barley genome on an ordinary personal computer. Some tools are highly sensitive to species-specific parameters. All produce false positive predictions and do not retrieve all known LTRs. Finally, only a few of these tools were designed with post-processing manual review in mind.

Thousands of plant genomes are being sequenced currently and in the near future. The 10KP Project for plant genomes (https://db.cngb.org/10kp/) and the Earth Biogenome Project (https://www.earthbiogenome.org) aim at sequencing a large number of plant genomes. This expansion of genomic data creates an urgent need for modern software tools to aid in detecting LTR-RTs in the new plant genomes; such tools should remedy the limitations of the currently available tools.

To this end, we have developed LtrDetector, which is a software tool for detecting LTR-RTs. LtrDetector depends on techniques inspired by signal processing. It is easy to install because it does not have any external dependencies. It can run on multiple machine cores in parallel, taking advantage of the advanced hardware available on personal computers. It is not species specific. It is more sensitive to known LTR-RTs than the related tools. It can process larger genomes such as the barley genome. It can produce images to facilitate the manual review/annotation of the newly located LTR-RTs.

Our efforts have resulted in the following contributions:

- The LtrDetector software for discovering LTR retrotransposons in assembled genomes. LtrDetector is available on GitHub (https://github.com/TulsaBioinformaticsToolsmith/LtrDetector) and in Additional file 1.
- Visualization script to view scores, which should aid in the manual verification of newly found elements — available in Additional file 1 and the GitHub repository.
- Novel pipeline to generate ground truth (sequences of known LTR retrotransposons). The pipeline is available in Additional file 2 and the GitHub repository.
- Putative LTR retrotransposons of six plant genomes (Additional files 3, 4, 5, 6, 7, 8, and 9).

Comparing the performance of LtrDetector to the performances of other related tools demonstrates that LtrDetector is the best de-novo tool currently available for detecting LTR-RTs. These results were obtained on synthetic sequences and multiple genomes.

## Implementation

### Overview

We used a variety of computational techniques to perform de-novo signature-based discovery of Long terminal repeat (LTR) retrotransposons (LTR-RTs). Signature-based tools rely strictly on specific structural features of LTR-RTs, e.g. the presence of two flanking LTRs, without referring to the nucleotide sequences of known elements. The main contribution of this study is a software package called LtrDetector. The tool utilizes methods inspired by signal processing, using the distances between copies of k-mers — short nucleotide sequences of length k — to determine the location of LTRs.

**Fig. 1:**
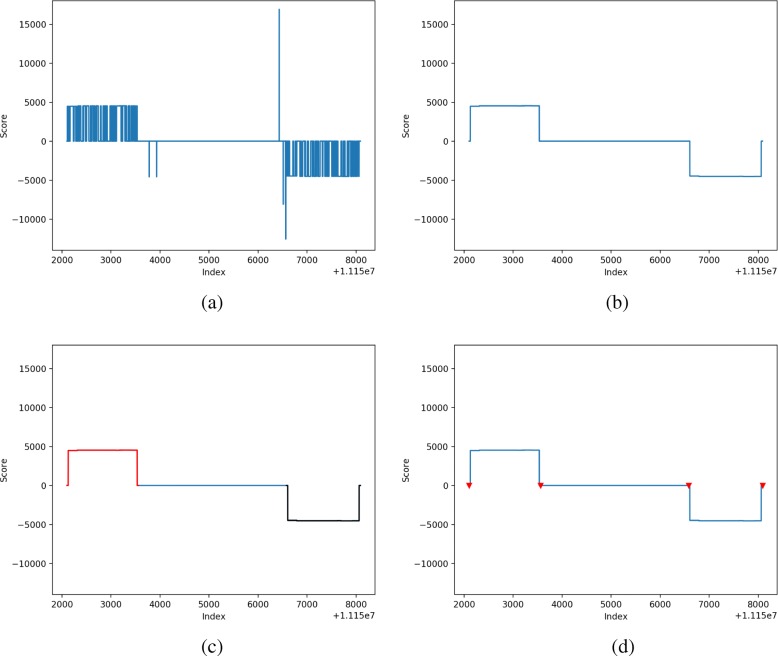
Method overview: LtrDetector is a software tool for locating long terminal repeat (LTR) retrotransposons (RTs). **a** A sequence of scores reflects the distance to the closest exact copy of the k-mer starting at each nucleotide. **b** Smoothed scores are produced after adjacent spikes are merged into a contiguous region. **c** Plateau regions are identified. Separate plateaus here are represented by black and red lines. **d** Plateaus are paired and their boundaries are adjusted. The red triangles denote the start and end coordinates for each LTR
- Processing the scores to merge adjacent stretches of similar scores (i.e. plateaus);
- Collecting plateaus and pairing those whose distance scores point to each other;
- Correcting LTR coordinates via local alignment of the regions surrounding each plateau in a pair; and
- Removing faulty candidates based on sequence identity, element length, and structural similarity to other types of transposable elements (TEs).

At a high level, LtrDetector locates LTR-RTs using the following steps (Fig. 1):

- Mapping each nucleotide in a sequence to a positive or negative numerical score recording the distance to the closest exact copy of the k-mer starting at that nucleotide;

### Scoring the input sequence

At the time of insertion into the genome, the two LTRs of an LTR-RT will be identical [4]. Centuries worth of mutation will lead to some degeneration, but the LTRs should retain a high degree of homology.

The goal of the scoring step is to mark the genomic distance to the nearest exact copy of every k-mer in the input sequence using a data-structure called a hash-table. A hash-table is conceptually similar to a dictionary, containing entries mapping a unique key to an associated value. It employs a mathematical function called hashing to convert a key into an index, which is used to look up the value in the hash-table’s underlying array.

LtrDetector utilizes a hashing function specific to DNA sequences. Each nucleotide (A, C, G, and T) in a k-mer is encoded as a digit (0, 1, 2, and 3). This digit sequence is considered as a quaternary (base-4) number and converted to a decimal number (base-10) that indicates an index within the array. Horner’s rule is used for efficiently converting the number from its quaternary to its decimal representation [55]. We have used similar data structures successfully in other software tools [56–59]. For example, the 5-mer ACCTG is transformed to 01132 (base 4) and then to 94 (base 10), mapping it to the 94^*th*^ cell in the array. Note that the array for storing all k-mers will be of length 4^*k*^.

LtrDetector traverses the input sequence nucleotide by nucleotide, computing the index for the k-mer starting at each position. As it encounters a particular k-mer for the first time, it will fill in the hash-table value with the initial location. Whenever that k-mer is found again, it will update the hash table with the new location and report the score at that index as the distance between the current copy and the previous copy.

Distances to and directions of the closest copies are recorded both forward and backward in the genome as positive and negative numbers. This process requires only one pass through the sequence because the direction of the closest copy can be calculated from the index of the k-mer and the index of the closest copy that is stored in the hash table. Scores will be updated if a copy is found closer downstream. The distance between the k-mer and its copy must be within a specific range due to the length properties of LTR retrotransposons [1].

### Processing scores

**Fig. 2:**
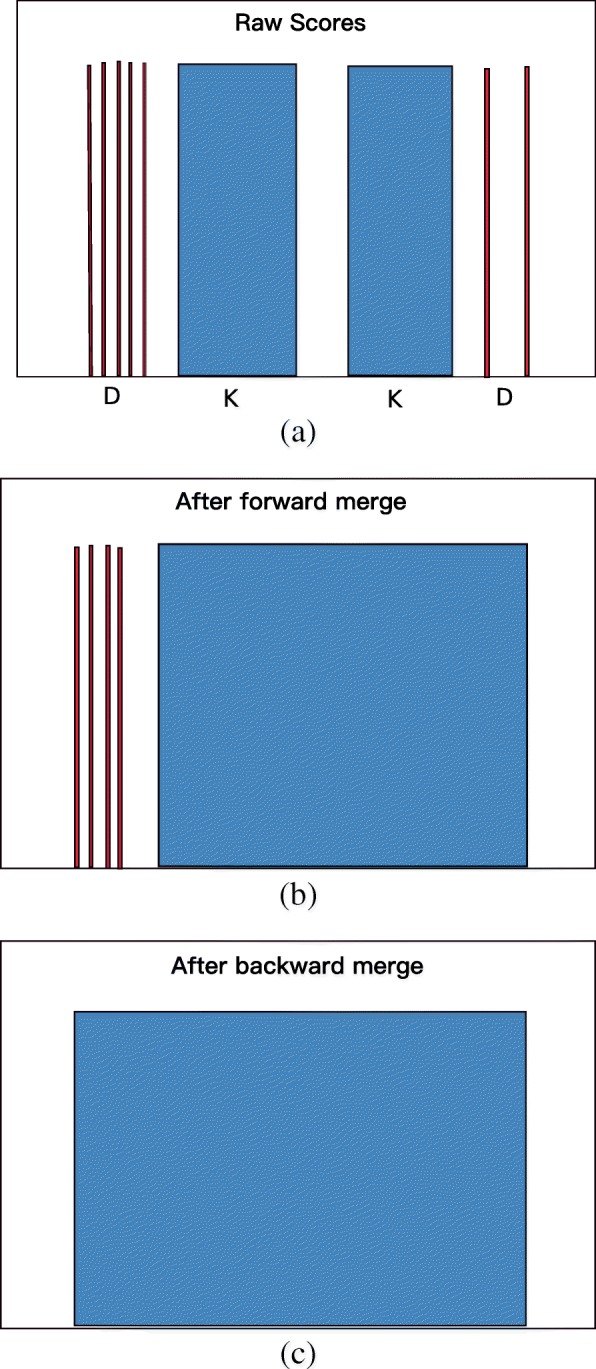
(**a**) Contiguous stretches of the same non-zero score are identified and marked as keep (K) or delete (D). (**b**) The forward pass merges K sections toward each other and adjacent D sections. (**c**) The backward pass merges remaining D sections that are close to K sections

The raw scores yielded by the previous step are processed to accentuate meaningful patterns. Wherever there is a significant repeat in the genome, there should be an extended, semi-continuous sequence of similar scores. However, any mutation will cause gaps in these stretches. LtrDetector first identifies all continuous stretches of non-zero scores, categorizing them as “keep” — K — if they are longer than or equal to a minimum seed value, (default: 10 bp), or “delete” — D — if they are not. The forward merging step merges a D section with a neighboring K section if the two are separated by a gap of less than a certain size (default: 200 bp). To merge, the scores belonging to the D section are overwritten with the median score of the adjacent K section, as are the scores in between. This D section is re-categorized as a K. Neighboring K sections will be merged by re-scoring only the gap section, using the median score of one of the two K sections. Next, the backward merging step proceeds in the opposite direction, merging all D sections that appear upstream of K sections and are missed by the forward merging step. After both passes, all remaining D selections are overwritten to zero to reduce noise. We illustrate this procedure in Fig. 2.

### Pairing plateaus

The merging step should produce wide plateaus in the scoring signal. The magnitude of their scores can be thought of both as the height of the plateau and as the distance towards its match. The sign of the scores indicate the direction — positive for downstream and negative for upstream. For instance, a plateau of width 200 and height +8000 should imply a similarly wide plateau of height -8000 starting about 8000 base pairs downstream. In this way, the scores of the two plateaus point towards each other.

Another hash-table-like data structure helps pair matching plateaus to form a full retrotransposon. Plateaus are assigned to a bin based on the magnitude of their height, with each bin holding plateaus within a certain range of height. The algorithm then steps through the candidates, placing each positive plateau into the appropriate bin as it is encountered. Each negative plateau will be assigned an initial bin, which will be searched for a positively-scored plateau located at the proper distance; recall that this distance is implied by the height of the negative plateau. Because we allow for some difference in height, the bins immediately above and below the initial one may be inspected. If a match is found, the two regions are returned and listed as a candidate LTR pair. If not, the negative plateau is discarded.

### Boundary correction

The newly paired LTR candidates merely approximate the boundaries of a putative retrotransposon because of the sensitivity of k-mers to mutations. Next, we use the Smith-Waterman local alignment algorithm [60] to sharpen the LTR boundaries.

As the plateaus may be of unequal length at this stage, we define a value L equivalent to the length of the larger plateau. From the center of each plateau, we mark a window of size 1.5×*L* bp in each direction. The resulting windows may not be longer than the maximum LTR length parameter (6000 bp is the default). We align these two regions, and the returned alignment indicates the corrected boundaries for the putative LTR-RT. Alignment identity scores are stored for later use. Scaling the alignment window based on the initial plateau length provides for good average case run time while still allowing LtrDetector to discover elements with large LTRs.

### Filtering

Several filters are applied to reduce the number of false positives. LTR identity scores from the previous step are used for discarding all entries whose paired LTRs exhibit sequence similarity below a given threshold (default: 85%). Then elements are filtered by size to remove those where either the LTR or the whole element is too small or too large, using values typical of known LTR-RTs. Our default range for the full element is 400–22000 and 100–6000 for its LTRs.

Next, the candidates are analyzed to determine whether they exhibit features of DNA transposons, which are another type of TE that appear in high copy number in many genomes. DNA transposons of the same family can appear in close proximity and be falsely identified as LTR-RTs by the previous steps. DNA transposons contain terminal inverted repeats, meaning that the reverse complement of the beginning sequence appears at the end of the element. LtrDetector locally aligns the first 30 nucleotides of each LTR with the reverse complement of its last 30 nucleotides. If this resulting alignment is sufficiently long (>15 bp), the element may represent two DNA transposons within close distance to each other; this element is discarded.

### Structural Annotations

Other structural features — Target Site Duplication (TSD) and Polypurine Tract (PPT), and the TG..CA motif are included as annotations.

A TSD is a small exact repeat that may occur at the insertion site. LtrDetector searches for TSDs using the longest common substring algorithm, which is a dynamic programming algorithm that finds exactly matching substrings of two strings. We run this algorithm on the regions consisting of the 20 bp before the left LTR and after the right LTR. The tool finds the closest TSD of at least 4 bp — if one exists.

Additionally, LtrDetector searches for a PPT, which is a region of highly enriched purine (A and G) content that appears in the interior region immediately adjacent to the 3’ LTR. We calculate a search window based on the size of the interior region on the LTR-RT. The tool searches for a minimum length seed composed entirely of purines, then expands in both directions from the first such seed, allowing for gaps. When the maximum gap is exceeded, the length and the purine percentage of the putative PPT are calculated. If these values are below certain minimums, the algorithm proceeds to the next seed and repeats the process. This search continues until an acceptable PPT is found or the search window is exceeded. LtrDetector also searches for a PPT on the negative strand by scanning the reverse complement of the search window, giving a clue as to the orientation of the LTR-RT.

Finally, we search for the TG..CA box. We scan the first 20 bp of the LTR-RT for the first occurrence of the TG motif and the last 20 bp for the final occurrence of the CA motif. If both motifs occur, we report the start and end of the box as an alternative boundary for the full LTR-RT.

### Reporting

By default, LtrDetector reports results in a tabular format, including columns for the start and the end coordinates of each retrotransposon, its constituent LTRs, and any found TSD, PPT, or TG..CA Box. Alternatively, it can produce output in BED format for easy evaluation using comparison utilities like bedtools. In this case, the output columns contain start and end coordinates for the entire element and for its constituent LTRs.

### Visualizing putative LTR-RTs

We have built this tool with manual verification in mind. LtrDetector comes with a Python program to enable the user to visualize the scores — distances and directions — as well as the boundaries of the two LTRs. The visualization program produces graphs. The x-axis displays the nucleotide indexes, and the y-axis displays the forward/backward distances to the closest copies. Forward distances are represented as positive numbers, whereas backward distances are represented as negative numbers. Figure 1a shows the scores. The merged plateaus can also be visualized (Fig. 1b). The boundaries of each LTR are demarcated by two inverted, red triangles (Fig. 1d). Looking at these graphs, the user can quickly assess the quality of predicted elements by comparing the identified boundaries with the surrounding scores. This should provide important information for the manual review process.

### Ground truth generation

In order to assess the accuracy of our signature-based predictions, we built a pipeline for assembling ground truth using previously known sequences of LTR-RTs. Repbase is the most comprehensive database for repetitive elements, containing consensus sequences for a wide variety of genomes [61]. The Repbase browser system provides FASTA files containing the LTRs and interior sequences for full LTR-RTs separately. The ground truth were constructed using two related, complementary approaches.

In the first approach, we downloaded these files and parsed them to append the LTR sequences before and after their associated interior sequence to form full LTR-RT elements. We then performed a BLAST search for these complete elements against the input genome, processing the output from BLAST to accept only those results that represent 100% coverage of the query as well as 70% or more identity.

In the second approach, we built a pipeline around RepeatMasker — the standard database-driven tool for repeat identification. RepeatMasker also uses Repbase as its default source of repeat consensus sequences. Instead of concatenating the LTRs and their interior regions before the search, we searched for them separately in RepeatMasker’s output. These entries were used for extracting LTR-RT coordinates by finding two 100%-query-coverage regions of the same LTR that are 400–22000 bp apart as defined by their start coordinates. The corresponding interior element was required to appear somewhere in between and to have 70% query coverage.

The outputs of the two pipelines were merged and duplicates were removed. The reason we used both pipelines is that the results from the RepeatMasker pipeline are dependent upon the estimated length parameters, but do a better job finding LTR-RTs with more degenerate interior regions, whereas the BLAST data is free from guesswork but stricter about enforcing the canonical structure of LTR-RTs.

### False positive evaluation

We built a false positive detection pipeline by parsing RepeatMasker output to determine when putative LTR-RTs overlap with non-LTR repeats. RepeatMasker-reported elements that do not belong to an LTR (excluding simple and low-complexity repeats) are compared with the predicted LTR-RTs. If two repeats of the same type overlap by more than 80% with the two supposed LTRs of a putative LTR-RT, this element is considered a false positive. However, if other repetitive elements overlap the interior of the predicted LTR-RT, this putative element is not counted as a false positive because nested repeats are very common. This approach was inspired by another study for detecting Miniature Inverted-repeat Transposable Elements (MITEs) [62]; in that study, a putative element is considered a false positive if it overlaps with any non-MITE elements. Repbase is by no means a complete record of repetitive elements, so neither the ground truth nor the false positive annotations will be comprehensive. Accordingly, a large amount of elements discovered by LtrDetector will overlap with neither set and will be impossible to evaluate against existing databases. Nonetheless, this approach — in our opinion — is the best available method for evaluating the false positives of tools for discovering LTR-RTs.

### Evaluation measures

True Positives (TP) are the discoveries that overlap with an entry in the ground truth. False Negatives (FN) are elements listed in the ground truth but not found by a tool. False Positives (FP) are the discoveries that overlap with an entry in the false positive data set. Mutual overlap is required to be 95%; for example, sequences A and B are counted as equivalent if the overlapping segment between A and B constitutes 95% of both A and B. Because we calculate this overlap on the whole element, it is theoretically possible that this definition of overlap may overlook some slight inaccuracies in the length of the LTRs. We use the standard measures of sensitivity (Eq. 1) and precision (Eq. 2) to assess the performances of LtrDetector and the related tools. Sensitivity is the ratio (or percentage) of the true elements found by a tool, whereas precision is the ratio (or percentage) of the true elements identified by a tool to the total number of regions predicted by the same tool.

1 $$  Sensitivity = \frac{TP}{TP+FN}  $$

2 $$  Precision = \frac{TP}{TP+FP}  $$

Additionally, we report the F1 measure (Eq. 3), which combines sensitivity and precision.

3 $$  \begin{aligned} F1 & = 2 \times \frac{Precision \times Sensitivity}{Precision+Sensitivity}\\ & = \frac{2 TP}{2 TP + FP + FN}\\ \end{aligned}  $$

Comparing the F1 measure to the accuracy (Eq. 4) shows how similar these two measures are.

4 $$  Accuracy = \frac{TP + TN}{TP + TN + FP + FN}  $$

Here, TN stands for True Negatives — unknown in this study. Therefore, the accuracy cannot be calculated. If we substitute TN with TP in the accuracy equation, we obtain the F1 measure equation. In other words, the F1 can be viewed as an accuracy measure when the TN cannot be determined.

### Data

We validated the results of LtrDetector using a variety of genomes. An initial test replicated the experiment in a study by Lerat [1], testing multiple tools on the X Chromosome of the *D. melanogaster* (Dm3) against a ground-truth annotation assembled from RepeatMasker. We performed similar analysis on the following genomes:

- *Arabidopsis thaliana* (TAIR10): http://plants.ensembl.org/Arabidopsis_thaliana/Info/Index
- *Hordeum vulgare* (HvIbscPgsbV2): http://plants.ensembl.org/Hordeum_vulgare/Info/Index
- *Oryza sativa Japonica* (IRGSP1): http://plants.ensembl.org/Oryza_sativa/Info/Index
- *Sorghum bicolor* (SorghumBicolorV2): http://plants.ensembl.org/Sorghum_bicolor/Info/Index
- *Zea mays* (ZeaMaysAGPv4): http://ensembl.gramene.org/Zea_mays/Info/Index
- *Glycine max* (Gmax_109): http://www.plantgdb.org/XGDB/phplib/download.php?GDB=Gm

### Parameter defaults

We conducted an empirical analysis of the Repbase sequences for six genomes in order to set the element length parameters for LtrDetector and the other tools. Table 1 shows length statistics of LTR-RTs of these genomes. We chose the default values to include almost all elements found in the data. The range for LTR length is 100–6000 bp, and 400–22000 bp for whole LTR-RTs. The whole element maximums and minimums are also used in our ground truth generation.

**Table 1:** Length statistics to determine the default parameters of LtrDetector

		*A. thaliana*	*O. Sativa*	*G.max*	*S. bicolor*	*Z. mays*	*H. vulgare*
LTR length	Maximum	2033	5832	2886	5645	**6119**	5609
	Minimum	103	109	105	100	**97**	154
	Mean	495	1128	421	731	679	1840
	Standard deviation	436	1399	356	942	936	1674
Total length	Maximum	14069	20595	18868	**22029**	20354	16260
	Minimum	824	**402**	2565	764	536	5143
	Mean	5635	5982	5516	6697	6331	9449
	Standard deviation	2083	2995	1998	2979	2692	3274

These statistics were calculated on LTR-RTs of six plant genomes found in Repbase. In Repbase, an LTR-RT is reported as two sequences: the LTR sequence and the interior sequence. Default length parameters were chosen to approximate the most extreme values found in the dataset, which appear in boldface. To calculate the total length of an LTR-RT, we concatenated two LTR sequences to the two sides of its interior sequence

**Fig. 3:**
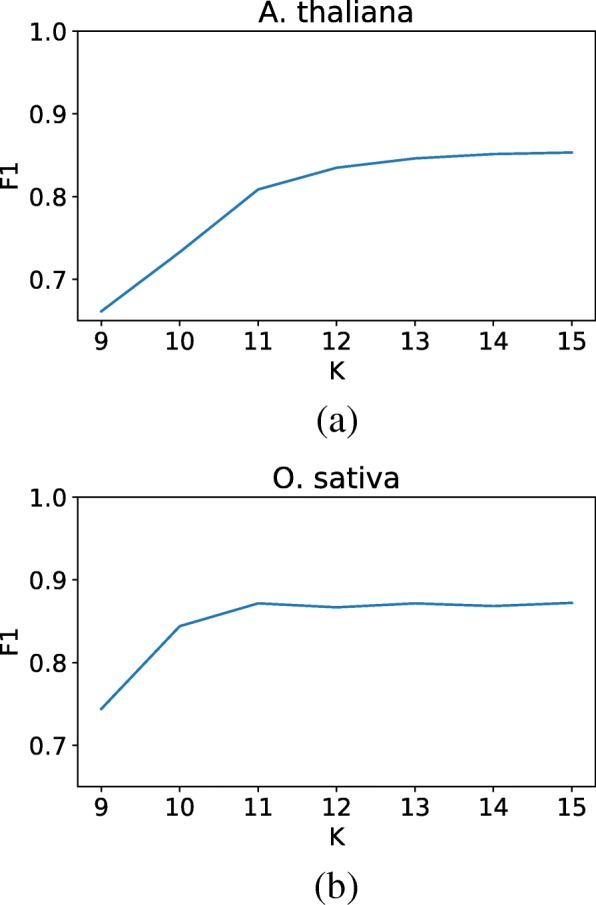
The effect of different values of k — the size of the short words, which are used as the keys in the hash table — on the F1 measure. As the value of k increases from 9 to 11 or 12, the F1 value increases (the higher, the better). The performance does not change markedly after that. (**a**) Shows the experiment on A. thaliana, (**b**) shows O. sativa

The value of k is extremely important to both the effectiveness and the efficiency of LtrDetector. If the chosen value is too small, many k-mers will occur by chance and the scores will contain a large amount of noise. If k is too large, the signal will likely miss more degenerate repeats. The memory usage is also proportional to the value of k; an increase of k by 1 increases the size of the hash table 4-fold. We evaluated LtrDetector on both *A. thaliana* and *O. sativa* for all values of k between 9 and 15 inclusive, and tracked the performance of each trial on F1. Figure 3 displays these results. On the basis of this experiment, we selected 13 as a suitable default k value because it provides excellent performance while keeping memory usage moderate. Users who are particularly concerned with memory may want to select a smaller k; a higher k may produce slightly better results at the cost of memory.

## Results and discussion

### Results on the X chromosome of the *Drosophila melanogaster*

Our initial test is based on the experiment by Lerat [1]. Table 2 shows the performances of these four tools: LTR_finder, LTR_seq, LTRharvest, and LtrDetector. All are evaluated using their default parameters. Although other tools like LTR_STRUC and MGEScan-LTR exist to discover LTR-RTs, they all had issues with availability and/or installation, so we were unable to get them to produce results. LtrDetector finds one fewer element that LTRharvest (92/96 vs. 93/96), while making 20% fewer total predictions (160 vs. 200). LTR_seq performed the worst of the tools on every metric, and will be excluded from further experiments. These results are an early indication that LtrDetector performs well relative to the currently available tools. *D. melanogaster* has a small genomic size and extremely well-preserved LTR sequences, making this a relatively easy test. Further evaluations are necessary to accurately gauge the performance of any tool.

**Table 2:** Results on the X Chromosome of *D. melanogaster*: We evaluated four de-novo tools on a ground-truth annotation provided by Lerat [1]

	Total	TP	Sensitivity	Memory	Time
Tool		Of 96	%	MB	sec.
LTR_Finder	57	48	50.0	300.3	390
LTR_seq	204	48	50.0	874.4	7262
LTRharvest	200	93	96.9	190.5	23
LtrDetector	160	92	95.3	1209.3	15

Total is the number of proposed LTR-RTs, TP stands for true positives

### Results on synthetic data sets

We built synthetic data by randomly generating exact repeats — long terminal repeats — within a certain size range, mutating a selected percentage of one of them, and inserting them with random sequences in between. Although this data set does not accurately simulate the content of a real genome, it can help us demonstrate the ability of a tool to discover repeats at a given level of mutation (See Table 3). LTR_Finder is one of the best-performing predecessor tools to LtrDetector, but its results are not listed because its strict filtering system requires other structural features that our synthetic genome lacks. For each trial, both tools are run with a sequence identity threshold of 5% lower than the similarity implied by the mutation rate. For example, the trial with 15% mutation would have an identity threshold of 80%. All other parameters are left at their defaults. Both tools capture nearly all of the elements in well conserved repeats (0–5%), but by 15% mutation, LtrDetector identifies 74 of 92 ground truth elements, whereas LTRharvest finds only 29. On the 20% mutation rate, LtrDetector outperformed LTRharvest by a wide margin in terms of the sensitivity (40/93 vs. 8/93). Neither tool is capable of reliably detecting repeats at 30% or greater mutation rates. These results are indicative of LtrDetector’s capabilities on repeats of varying levels of degeneration.

**Table 3:** Results on synthetic genomes: We constructed several synthetic chromsomes with randomly generated direct repeats mutated at a given percentage of nucleotides (0–30%) to assess performance at different levels of LTR conservation

Tool	Total	TP	GT
*Mutation 0%*			
LTRharvest	90	90	90
LtrDetector	90	90	90
*Mutation 5%*			
LTRharvest	92	91	92
LtrDetector	90	90	92
*Mutation 10%*			
LTRharvest	78	74	91
LtrDetector	88	88	91
*Mutation 15%*			
LTRharvest	32	29	92
LtrDetector	74	74	92
*Mutation 20%*			
LTRharvest	10	8	93
LtrDetector	40	40	93
*Mutation 30%*			
LTRharvest	1	0	90
LtrDetector	2	2	90

Total is the number of proposed LTR-RTs, TP is number of true positives, GT is number of elements in the synthetic ground truth

### Results on six plant genomes

Our main experiment was an evaluation of three tools (LtrDetector, LTR_Finder and LTRharvest) on six plant genomes (including several important crops) of varying size and repeat content.

In this experiment, all tools were run using parameters determined on sequences found in Repbase (see Table 4). Results for LTR_Finder are unavailable for the *Hordeum vulgare* (barley) genome because memory demands repeatedly caused the computer to crash on four computer cores, and a subsequent trial on one core was unable to finish over two weeks of run time (2/7 chromosomes finished). The results of this experiment suggest substantial performance gains for our tool over previous methods.

**Table 4:** Results on six plant genomes: We tested three tools on one model organism, *A. thaliana*, and five important crops of varying genomic size and repeat content

Tool	Total	TP	GT	FP	Sensitivity	Precision	F1	Time (hr:min:sec)	Memory (GB)
*A. thaliana*									
LTR_Finder	399	106	248	0	0.427	1.000	0.599	0:30:46	0.86
LTRharvest	2301	180	248	6	0.726	0.968	0.829	0:01:08	0.24
LtrDetector	1714	187	248	9	0.754	0.954	0.842	0:04:02	4.45
*O. sativa*									
LTR_Finder	5324	1163	1760	14	0.661	0.988	0.792	5:19:03	0.95
LTRharvest	9761	1392	1760	182	0.791	0.884	0.835	0:03:09	0.34
LtrDetector	7343	1442	1760	119	0.819	0.924	0.868	0:15:30	5.31
*S. bicolor*									
LTR_Finder	11734	4219	6565	67	0.643	0.984	0.778	10:43:26	1.62
LTRharvest	22700	4476	6565	502	0.682	0.899	0.776	0:04:23	0.60
LtrDetector	24682	5285	6565	214	0.805	0.961	0.876	1:10:47	6.1
*G. max*									
LTR_Finder	12141	1748	3130	7	0.558	0.996	0.716	25:18:21	1.88
LTRharvest	29016	2171	3130	20	0.694	0.991	0.816	0:09:35	0.48
LtrDetector	25537	2542	3130	12	0.812	0.995	0.894	0:43:06	6.11
*Z. mays*									
LTR_Finder	60860	11411	16839	13	0.678	0.999	0.807	111:21:37	12.36
LTRharvest	101943	11244	16839	102	0.668	0.991	0.798	0:15:00	2.36
LtrDetector	116923	13122	16839	71	0.779	0.995	0.874	5:53:08	9.62
H. vulgare									
LTR_Finder	–	–	–	–	–	–	–	–	–
LTRharvest	207016	4378	9164	492	0.478	0.899	0.624	1:33:29 *	5.12
LtrDetector	213367	6824	9164	199	0.745	0.972	0.843	17:24:04**	14.15
Total (Excluding *H. vulgare*)									
LTR_Finder	90458	18647	28542	101	0.653	0.995	0.789	153:13:13	–
LTRharvest	165721	19463	28542	812	0.682	0.960	0.797	0:33:15	–
LtrDetector	176197	22578	28542	425	0.791	0.982	0.876	8:06:33	–
Total (Including *H. vulgare*)									
LTRharvest	372737	23841	37706	1304	0.632	0.948	0.759	02:06:44	–
LtrDetector	389564	29402	37706	624	0.780	0.979	0.868	25:30:37	–

Parameters used for each tool can be found in the “Implementation” section. We used an additional utility to process each of LTR_Finder and LTRharvest in parallel because neither supports multi-threading. We did so to ensure fair comparison in terms of time since our tool, LtrDetector, is concurrent by default. Total is the number of proposed LTR-RTs, TP is number of true positives, GT is number of elements in the ground truth, FP are false positives. Sensitivity, Precision, and F1 are defined by Eqs. 1, 2, and 3. We report all measures for each genome and in total. Note: Results for LTR_Finder are unavailable for the *Hordeum vulgare* (barley) genome because memory demands repeatedly caused the computer to crash on four computer cores, and a subsequent trial on one core was unable to finish over two weeks of run time (2/7 chromosomes finished). All trials run on four cores unless otherwise noted. * LTRharvest run on one thread for *H. vulgare*. ** LtrDetector run on three threads for *H. vulgare*

Aggregate sensitivity (excluding the *H. vulgare* genome) is the classification measure in which we saw the most improvement, with our software tool identifying 79.1% of known LTR-RT overall, in comparison to 65.3% by LTR_Finder (improvement of 21.1%) and 68.2% by LTRharvest (improvement of 16.0%). When considering the aggregate sensitivity on the six genomes, LtrDetector outperformed LTRharvest (improvement of 23.4%). Additionally, LtrDetector produced fairly consistent results across the different genomes we tested, ranging from 74.5% on *H. vulgare* to 81.9% on the smaller *O. sativa*. LtrDetector was the most sensitive tool on all six genomes.

LTR_Finder predicted very few false positives and was the most precise tool overall at 99.5%. LtrDetector came in second at 98.2% followed by LTRharvest at 96.0%. All three tools found many more true positives than false positives, resulting in high precision overall.

On the F1 composite measure (excluding the *H. vulgare* genome), LtrDetector again achieves the highest score, outperforming LTR_Finder by 11.0% (87.6 vs. 78.9) and LTRharvest by 9.9% (87.6 vs. 79.7). When the *H. vulgare* genome is included, LtrDetector showed improvement of 14.4% over LTRharvest. These results demonstrate that LtrDetector strikes a balance between thorough collection of known LTR-RTs and avoiding spurious predictions.

Our evaluation criteria are dependent on the consensus sequences available in Repbase, so we will not be able to definitively classify the majority of putative LTR-RTs as true positives or false positives. Such elements will be unconfirmed, but could potentially be novel discoveries. On the first five genomes (excluding barley), LtrDetector and LTRharvest propose similar numbers of retrotransposons — 176197 and 165721, respectively. LTR_Finder is more conservative with only 90458 discovered elements.

The total number of identified elements helps in deriving an estimate of the percentage of a given genome that is composed of full-length LTR-RTs. We summed the length of each discovery in base pairs and divided this total by the number of base pairs in the entire genome. This produced estimates of 10.6% LTR-RT content for *A. thaliana*, 18.5% for *O. Sativa*, 25.9% for *G. max*, 39.2% for *S. bicolor*, 48.2% for *H. Vulgare*, and 62.4% for *Z. mays*.

The three tools have vastly different run times. LtrDetector can work on multiple FASTA files in parallel, whereas we had to configure the other two tools to process several chromosomes simultaneously using the GNU parallel command utility. The experiments were run on all four cores of an Intel i5 machine with 16 GB RAM running Ubuntu. We recorded wall-clock time using the Linux time command. LTRharvest was by far the fastest tool, capable of processing the five smallest genomes in just over 33 min. On the other end of the spectrum, LTR_Finder took about 153 h — more than 6 days. LtrDetector’s runtime efficiency was in the middle (around 8 h).

LTRharvest uses far less memory overall. LTR_Finder requires moderate memory on the small genomes. LtrDetector consistently had the highest memory requirements of the the three tools.

The above experiments suggest that LtrDetector represents a substantial advance in the available methods for discovering LTR-RT elements de-novo. In comparison to related software tools, it delivers more accurate predictions in reasonable time using memory readily available on modern personal computers. Its capabilities are proven not only on simple model organisms but also on a wide variety of plant genomes.

Crucially for researchers, the tool is easy to install and run and will perform well on an ordinary desktop computer. It provides a robust set of default parameters for maximum generality, but still allows for user configuration via command-line options. As more genome sequences become available, the utility of tools like LtrDector will only increase.

### Gene validation

We obtained protein sequences for the *gag/pol* genes from the UniProt database and used tBLASTn (protein to nucleotide) to search for them inside of our predictions. We recorded the percentage that exhibited more than 25% query coverage. The results for the ground truth dataset and the predictions by LTR_Finder, LTRharvest, and LtrDetector are found in Table 5. For instance, the 24.9% of the identified elements with *gag/pol* for LtrDetector on *G. max* compares favorably with the 19.7% for LTR_Finder and 17.6% for LTRharvest. LTR_Finder’s putative LTR-RTs contain slightly more genes on *A. thaliana* (49.1% to LtrDetector’s 48.0%). LtrDetector’s low rate of 10.4% on *S. bicolor* is in line with the 9.0–11.0% from the ground truth and the other two tools. Even with our strict ground truth generation (at least 70% of the interior of an LTR-RT is required to be covered at 70% or more identity), the proteins did not consistently appear in this ground truth data set. The sequences gag/pol are the best available on the UniProt database, but we stress that they are unconfirmed and should only be taken as a primitive sign of the biological relevance of all predictions. These results show that LtrDetector’s putative LTR-RTs are enriched with fragments of these two genes, suggesting the quality of elements identified by LtrDetector relative to those predicted by the other tools.

**Table 5:** Gene content validation: We searched for species-specific fused gag/pol in the interior of the known and the predicted LTR-RTs

	*A. thaliana*	*O. sativa*	*G. max*	*S. bicolor*
Ground truth	0.65	0.43	0.51	0.10
LtrDetector	0.48	0.31	0.25	0.10
LTR_Finder	0.49	0.28	0.20	0.11
LTRharvest	0.35	0.21	0.18	0.09

### Nested element discovery

Although this feature was not enabled for the above analysis, the current version of the software includes beta functionality for finding nested LTR-RTs. The first pass of LtrDetector discovers non-nested elements and nested elements that are small enough to meet the length requirements of LTR-RTs. Optionally, it conducts an equivalent search around each discovered element, automatically adjusting the scoring system parameters to identify elements that fully enclose the elements discovered in the first pass.

### Post-processing manual annotation aid

LtrDetector is unique in providing a simple visualization tool to aid with manual verification of putative LTR-RTs. For each LTR-RT identified by LtrDetecor, the script will produce a colorful graph showing distances between k-mers (short words of length k) and their nearest copies as well as markers for the start and end locations of each LTR. See Fig. 1 for examples. This signal will ideally show two flat plateaus representing two LTRs.

### Comparisons to related tools

LtrDetector represents an innovative approach to repeat discovery that differs greatly from its predecessor tools. LtrDetector uses techniques inspired by signal processing. The use of a signal of k-mer distances as an indication of repeat locations is the first of its kind. Both of our closest competitor tools use suffix-arrays, which are complex data structures that have been widely used in text processing [63]. LTRharvest uses a suffix-array to identifies initial maximal repeats — seeds — and a greedy dynamic programming algorithm called X-drop extension to expand from the seeds [51]. It can filter based on length, LTR identity, target site duplications, and the palindromic LTR motif (i.e. TG..CA box). LTR_Finder begins with all sets of exact repeats found by the suffix-array [50]. Each member in every set is considered in a pair-wise fashion. The region between the two start coordinates in a pair is aligned with the region between the two end coordinates. The pairs are merged if the alignment is above a certain threshold. Similarly to LtrDetector, LTR_Finder uses the Smith-Waterman local alignment algorithm [60] for boundary adjustment. LTR_Finder concludes with an aggressive filtering system based on searching for target site duplications, the TG..CA box, primary binding sites, and the proper protein domains in the interior sequence.

### Future work

Future work will seek to improve time efficiency, largely by reducing our dependence on local alignment, which is very slow on longer sequences. This may include replacing the Smith-Waterman algorithm with more efficient approximations. We will seek to reduce memory consumption by optimizing the C++ code-base and developing an iterative approach that will allow LtrDetector to sequentially load pieces of larger chromosomes from storage. We will add the option to use the structural features (TSD etc) as filters rather than just annotations. We will also work to improve the beta version of the nested LTR discovery that is included with the software.

## Conclusions

In this study, we developed and tested a software tool called LtrDetector, which identifies Long Terminal Repeat Retrotransposons (LTR-RTs) de novo in assembled genomes. Our software addresses some of the scaleability and usability concerns of older tools and is better capable of matching the performance of tools that leverage consensus sequnces. LtrDetector revolves around a novel repeat detection methodology that calculates k-mer distance scores to recover underlying repeats. It supplements this with an alignment-based correction and filters to enforce the structure of LTR-RTs. This methodology provides accurate predictions across a diverse range of input genomes. Using consensus sequence predictions from six plant genomes, including maize and barley, we proved that our tool is significantly more sensitive than the previous two most successful software tools, LTR_Finder and LTRharvest.

We believe that LtrDetector can provide valuable computational support to researchers, particularly those studying plant genomes. It reports biologically relevant features of the LTR-RTs and includes a k-mer score visualization script to aid with manual review. It is simple to use and performs well on an ordinary personal computer. As the number of sequenced genomes increases by the day, the potential impact of LtrDetector also increases. Automated, accurate identification of LTR-RTs will enable researchers to further investigate the regulatory capacities of LTR-RTs, and could hold great promise in understanding plant evolution and crop productivity.

## Availability and requirements

The source code (C++ and Python) is available as Additional file 1.

**Project name:** LtrDetector.

**Project home page:** https://github.com/TulsaBioinformaticsToolsmith/LtrDetector

**Operating system(s):** UNIX/Linux/Mac.

**Programming language:** C++ and Python.

**Other requirements:** BLAST (https://blast.ncbi.nlm.nih.gov/Blast.cgi) and Bedtools (http://bedtools.readthedocs.io/en/latest/). Python: NumPy, Matplotlib, Pandas.

**License:** The software is provided as-is under the GNU GPLv3.

**Any restrictions to use by non-academics:** License needed.

## Additional files

Additional file 1 The LtrDetector software and the visualization script. This compressed file (.tar.gz) includes the C++ source code of LtrDetector and the Python script for visualizing putative elements as well as instructions on how to compile and run the programs. (TAR.GZ 145 kb)

Additional file 2 Script for ground truth generation pipeline. This compressed file (.tar.gz) includes the Python code for the evaluation pipeline. (TAR.GZ 5 kb)

Additional file 3 The synthetic sequences. This compressed file (.tar.gz) includes the synthetic sequences with different mutation rates. (TAR.GZ 1872 kb)

Additional file 4 Long Terminal Repeat (LTR) retrotransposons found by LtrDetector in *Arabidopsis thaliana*. This compressed file (.tar.gz) includes the LTR retrotransposons found by LtrDetector in BED format. (TAR.GZ 62 kb)

Additional file 5 LTR retrotransposons found by LtrDetector in *Glycine max*. This compressed file (.tar.gz) includes the LTR retrotransposons found by LtrDetector in BED format. (TAR.GZ 929 kb)

Additional file 6 LTR retrotransposons found by LtrDetector in *Hordeum vulgare*. This compressed file (.tar.gz) includes the LTR retrotransposons found by LtrDetector in BED format. (TAR.GZ 7488 kb)

Additional file 7 LTR retrotransposons found by LtrDetector in *Oryza sativa Japonica*. This compressed file (.tar.gz) includes the LTR retrotransposons found by LtrDetector in BED format. (TAR.GZ 268 kb)

Additional file 8 LTR retrotransposons found by LtrDetector in *Sorghum bicolor*. This compressed file (.tar.gz) includes the LTR retrotransposons found by LtrDetector in BED format. (TAR.GZ 891 kb)

Additional file 9 LTR retrotransposons found by LtrDetector in *Zea mays*. This compressed file (.tar.gz) includes the LTR retrotransposons found by LtrDetector in BED format. (TAR.GZ 4278 kb)

## Abbreviations

bp: Base pairs
LTR-RT: Long terminal repeat retrotransposon
LTR: Long terminal repeat
RT: Retrotransposon
TE: Transposable element
